# Blunted natriuretic response to saline loading in sheep with hypertensive kidney disease following radiofrequency catheter-based renal denervation

**DOI:** 10.1038/s41598-021-94221-5

**Published:** 2021-07-20

**Authors:** Reetu R. Singh, Zoe McArdle, Harshil Singh, Lindsea C. Booth, Clive N. May, Geoffrey A. Head, Karen M. Moritz, Markus P. Schlaich, Kate M. Denton

**Affiliations:** 1 Cardiovascular Program, Monash Biomedicine Discovery Institute and Department of Physiology, Monash University, Clayton, Melbourne, VIC 3800 Australia; 2 The Florey Institute of Neuroscience and Mental Health, The University of Melbourne, Parkville, Australia; 3 Baker IDI Heart and Diabetes Institute, Melbourne, Australia; 4 School of Biomedical Sciences, The University of Queensland, Brisbane, Australia; 5 School of Medicine and Pharmacology-Royal Perth Hospital Unit, University of Western Australia, Perth, Australia

**Keywords:** Physiology, Nephrology

## Abstract

Renal sympathetic nerves contribute to renal excretory function during volume expansion. We hypothesized that intact renal innervation is required for excretion of a fluid/electrolyte load in hypertensive chronic kidney disease (CKD) and normotensive healthy settings. Blood pressure, kidney hemodynamic and excretory response to 180 min of isotonic saline loading (0.13 ml/kg/min) were examined in female normotensive (control) and hypertensive CKD sheep at 2 and 11 months after sham (control-intact, CKD-intact) or radiofrequency catheter-based RDN (control-RDN, CKD-RDN) procedure. Basal blood pressure was ~ 7 to 9 mmHg lower at 2, and 11 months in CKD-RDN compared with CKD-intact sheep. Saline loading did not alter glomerular filtration rate in any group. At 2 months, in response to saline loading, total urine and sodium excretion were ~ 40 to 50% less, in control-RDN and CKD-RDN than intact groups. At 11 months, the natriuretic and diuretic response to saline loading were similar between control-intact, control-RDN and CKD-intact groups but sodium excretion was ~ 42% less in CKD-RDN compared with CKD-intact at this time-point. These findings indicate that chronic withdrawal of basal renal sympathetic activity impairs fluid/electrolyte excretion during volume expansion. Clinically, a reduced ability to excrete a saline load following RDN may contribute to disturbances in body fluid balance in hypertensive CKD.

## Introduction

Regulation of fluid and electrolyte homeostasis by the kidneys is a major determinant of long-term arterial pressure^1^. Impairments in kidney tubular sodium handling resulting in sodium retention are observed in animal models of hypertension^1^. It is suggested that overactivity of the renal sympathetic nerves may contribute to enhanced tubular sodium reabsorption and this may drive elevations in blood pressure^2,3^. In line with this, the blood pressure lowering effect of renal denervation (RDN) in hypertension is in part, attributed to a decrease in kidney tubular sodium reabsorption^2–4^. An increase in urinary sodium excretion following RDN has been observed in rats^1,5^, dogs^6^, rabbits^7^ and in hypertensive patients^8^.

However, RDN may impair electrolyte and fluid excretion in response to homeostatic challenge such as volume expansion. Resection of a kidney for kidney transplantation results in complete denervation. In a case study, natriuresis in response to acute volume expansion was shown to be blunted in the recipient between 6 and 10 months after renal transplant^9^. This observation was not confirmed in a later study in a recipient examined between 4 and 13 months after transplant^10^. Although not examined in the study, the authors suggested that reinnervation of renal nerves may underlie the inconsistent observations. In response to acute saline loading in conscious normotensive dogs^11^ or volume expansion by dextran in conscious normotensive monkeys^12^, excretion of sodium and water were blunted in denervated animals compared with non-denervated. In those studies, examinations in the animals were also conducted over a significantly varied period after RDN ranging from 1 to 23 days after procedure. In obese dogs with mild hypertension, RDN had no effect on sodium excretion after either an acute saline load^13^ or a sodium-rich meal^14^. However, in those studies, sodium excretion in response to acute saline load^13^ and a sodium rich meal^14^ were also blunted in intact obese animals. Therefore, renal nerves do not appear to play a significant role in excretion of a sodium load in obesity. In the normotensive conscious sheep, blunting of natriuretic and diuretic responses to hypertonic saline loading were observed acutely (1 week) after radiofrequency catheter-based RDN^15^. From these observations it cannot be deduced if renal nerves make an important contribution to excretion of a sodium and volume load. Given that RDN is clinically being trialed for treatment of hypertension^16–19^ and also chronic kidney disease (CKD)^3,20–24^, the observations demonstrating that RDN may promote fluid/electrolyte retention in response to volume expansion is concerning. Fluid retention in patients with impaired control of blood pressure and kidney function, such as those with hypertensive CKD, may result in adverse consequences. However, a major limitation of the studies mentioned is that, either the examinations have been performed in the short-term (acute studies) or because of the significant time-range post RDN, the findings are potentially influenced by reinnervation of the renal nerves. It is unclear from these studies whether the impairment in fluid/electrolyte excretion following RDN are a short-term defect, or if these changes are sustained over the long-term. To clearly understand the contribution of renal nerves and reinnervation of the renal nerves on excretion of a fluid and electrolyte load, it is important to examine whether RDN affects excretory function in the same animal long-term.

In normotensive sheep, following radiofrequency catheter-based RDN, partial structural and functional reinnervation of renal nerves is observed at 5.5 months after RDN and reinnervation is complete by 11 months after RDN^25^. Therefore, in the present study we examined fluid and electrolyte excretion in response to volume expansion in sheep at 2 months (period prior to reinnervation), and at 11 months (time of complete reinnervation) after radiofrequency catheter-based RDN. In this study, we included sheep with hypertensive CKD to understand the impact of RDN on fluid and electrolyte excretion in disease. In this ovine model, hypertensive CKD is generated by performing unilateral nephrectomy in the sheep fetus at 100 days of gestation (term = 150 days). Sheep thus born with a single kidney have elevated blood pressure, lower GFR, and albuminuria from 6 months of age and these exacerbate with ageing^26,27^. In this ovine model of hypertensive CKD, we have demonstrated enhanced vascular contraction to electrical stimulation of renal nerves and demonstrated hyperinnervation of both efferent renal sympathetic and afferent renal sensory nerves^28^. These observations support a role for elevated RSNA in this ovine model of hypertensive CKD. Additionally, radiofrequency catheter-based RDN successfully reduced blood pressure from 2 months after RDN, improved GFR and renal blood flow from 5 months after RDN and prevented albuminuria with effects sustained up until 30 months after RDN in sheep with hypertensive CKD compared with non-denervated counterparts^28,29^. These features make this ovine model of hypertensive CKD an appropriate model to examine the effects of RDN on renal excretory function over the long-term.

For this study we hypothesized that intact renal innervation is required for excretion of a fluid and electrolyte load during volume expansion in both normotensive and hypertensive settings. Renal excretory responses to 3 h of volume expansion via isotonic saline loading were examined in conscious normotensive sheep and in sheep with hypertensive CKD, at 2 and 11 months after sham (intact) or RDN procedure, using the single electrode *Symplicity Flex*^*®*^ radiofrequency catheter-based system.

## Results

### Time control series (vehicle infusion)

Arterial pressure, heart rate and renal hemodynamic (GFR, RBF, RVR and filtration fraction) did not vary markedly with time over the 7-h period of measurement (Data supplement, Figures S1a–d, S2a–h). Therefore, the response to saline loading was not compared with time-control series (vehicle infusion).

### Saline loading-arterial pressure and heart rate

**Figure 1:**
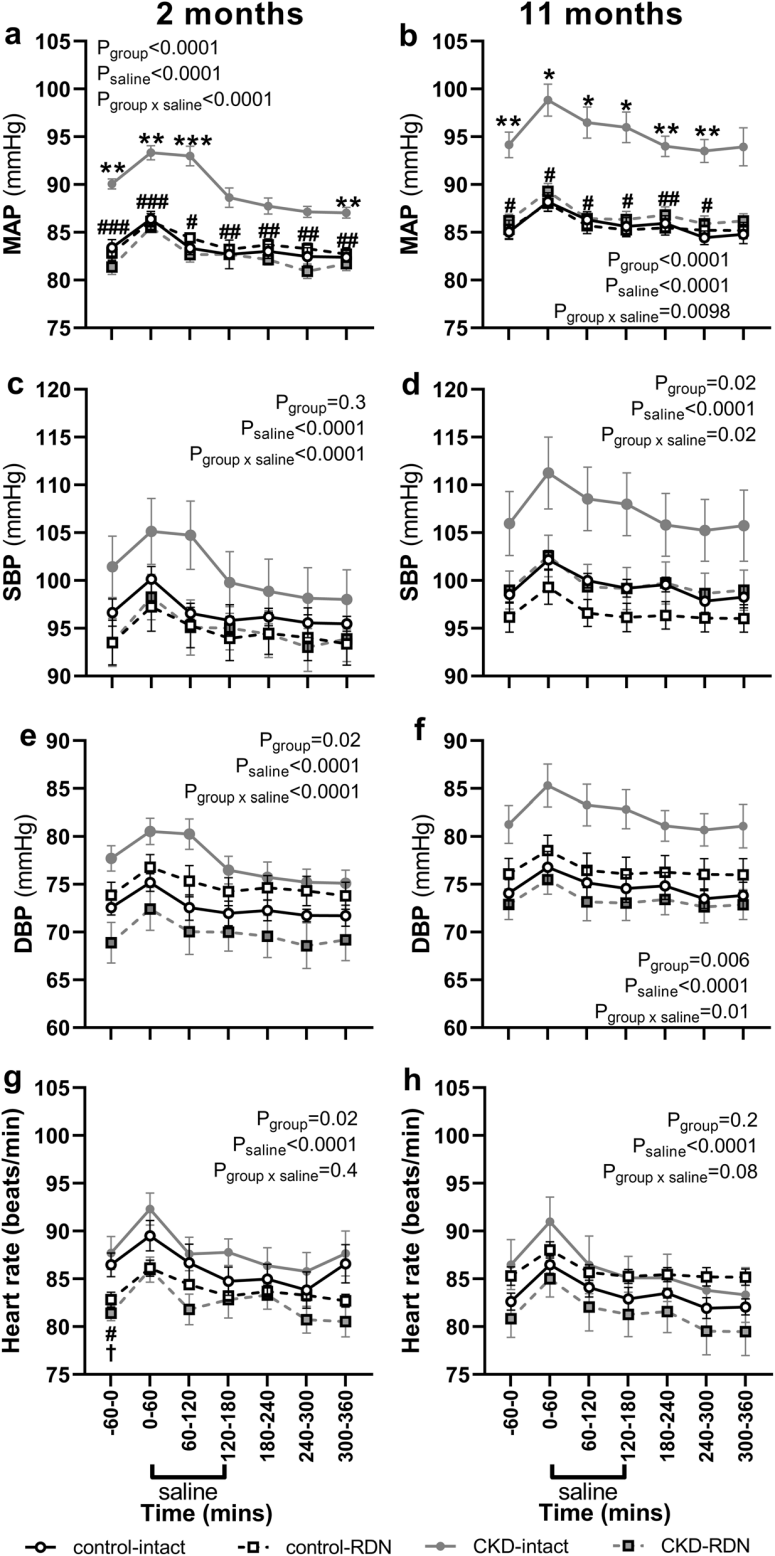
Arterial pressure and heart rate in response to isotonic saline loading. MAP; mean arterial pressure, SBP; systolic blood pressure, DBP; diastolic blood pressure. Data are at baseline; − 60–0 min, during 180 min of saline infusion and during 180 min of recovery post saline infusion. Responses were examined in normotensive and hypertensive CKD sheep at 2 and 11 months after sham (intact) or renal denervation (RDN) procedure. Data are mean ± SE. *P < 0.05, ***P < 0.001, ***P < 0.001 comparing control-intact with CKD-intact, ^#^P < 0.05, ^##^P < 0.01, ^###^P < 0.001 comparing CKD-intact with CKD-RDN, ^†^P < 0.05 comparing control-intact with control-RDN from post-hoc analysis following repeated measures ANOVA.

On the day of saline loading, at both time-points, basal MAP was higher in CKD-intact group compared with control-intact, and compared with CKD-RDN groups, but MAP was similar between control-intact and control-RDN groups (MAP mmHg; 2 months; CKD-intact: 90 ± 1, control-intact: 83 ± 1, P = 0.0006, CKD-RDN: 81 ± 1, P < 0.0001, control-RDN: 82 ± 1, 11 months; CKD-intact: 94 ± 1, control-intact: 85 ± 1, P = 0.0009, CKD-RDN: 86 ± 1, P = 0.002, control-RDN: 85 ± 1, Fig. 1a,b). MAP increased from baseline in response to saline loading in all groups (P_saline_ < 0.0001, Fig. 1a,b). The magnitude of increase in MAP was similar between groups at both time-points (~ 2 to 5 mmHg, Data Supplement, Figure S3a,b). Diastolic and systolic blood pressure response to saline loading were like that of MAP at both time-points (Fig. 1c–f).

Basal heart rate was similar between intact groups at both time-points (Fig. 1g,h). At 2 months after procedure, basal heart rate was lower in control-RDN (P = 0.05, Fig. 1g) and CKD-RDN (P = 0.03, Fig. 1g) groups compared with intact counterparts but not significantly different between RDN and intact groups at 11 months (Fig. 1h). In response to saline loading, heart rate increased similarly in all groups in the first hour of saline loading (~ 4 beats/min, P_saline_ < 0.0001) after which it returned to baseline levels (Fig. 1g,h, and data supplement, Figure S3c,d).

### Saline loading-plasma renin activity

**Figure 2:**
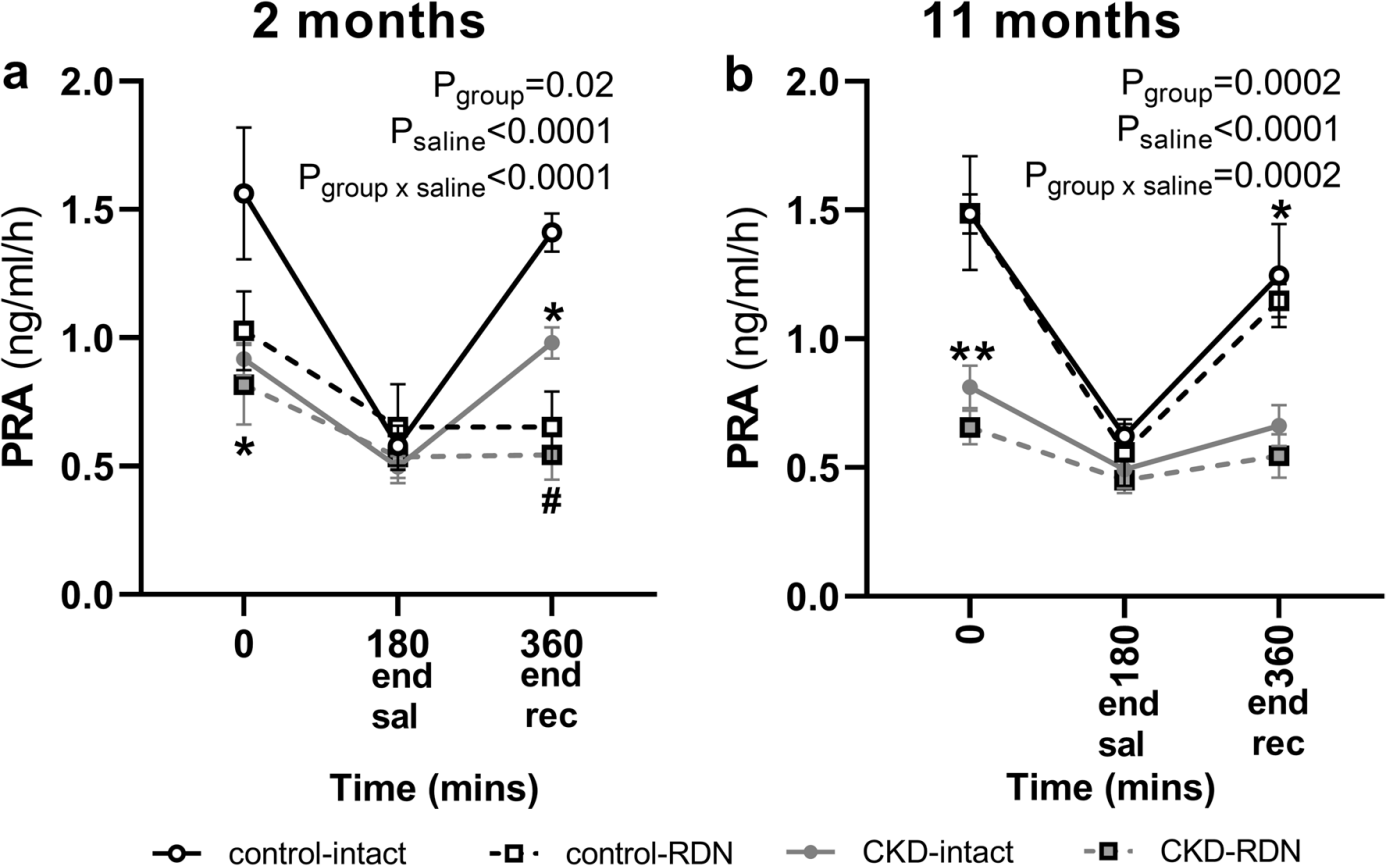
Plasma renin activity (PRA) in normotensive and hypertensive CKD sheep at 2 and 11 months after sham (intact) or renal denervation (RDN) procedure. Data presented at end of hour baseline (0 min), at the end of 180 min of saline infusion (end saline) and at the end of the 180 min recovery period post saline infusion (end rec). Data are mean ± SE. *P < 0.05, **P < 0.01 comparing control-intact with CKD-intact, ^#^P < 0.05 comparing CKD-intact with CKD-RDN from post-hoc analysis following repeated measures ANOVA.

Basal PRA was lower in CKD-intact sheep compared with control-intact at both time-points (2 months; ~ 41%, P = 0.004, 11 months; ~ 45%, P = 0.002, Fig. 2a,b). PRA was not statistically different between control-intact and control-RDN, and CKD-intact and CKD-RDN groups at either time-point (Fig. 2a,b). Between 2 and 11 months after RDN,PRA increased in control-RDN group (~ 45%, P = 0.008). In response to saline loading, PRA decreased from baseline (P_saline_ < 0.0001, Fig. 2a,b). The magnitude of fall in PRA during saline loading (expressed as percent change from baseline) was less in CKD-intact animals compared with control-intact at both time-points (2 months; P = 0.05, 11 months; P = 0.01, Table 1). In both RDN groups, the magnitude of fall in PRA during saline loading was less than intact counterparts at 2 months (control; P = 0.02, CKD; P = 0.05) but not at 11 months (Table 1). The fall in PRA was greater in control-RDN group at 11 months compared with 2 months (~ 28%, P = 0.003, Table 1).

**Table 1:** Total volume and sodium infused and excreted and change in plasma renin activity in response to isotonic saline loading.

	2 months post sham/RDN				11 months post sham/RDN			
	Control-intact (N = 6)	Control-RDN (N = 7)	CKD-intact (N = 7)	CKD-RDN (N = 7)	Control-intact (N = 6)	Control-RDN (N = 7)	CKD-intact (N = 7)	CKD-RDN (N = 7)
Body weight (kg)	31 ± 2	32 ± 2	30 ± 1	32 ± 2	41 ± 2^†††^	42 ± 1^†††^	39 ± 1^†††^	41 ± 1^†††^
Total volume infused (ml)	725 ± 47	756 ± 40	712 ± 26	757 ± 49	949 ± 37^†††^	975 ± 25^†††^	909 ± 24^†††^	956 ± 28^†††^
Total sodium infused (mmol)	112 ± 7	116 ± 6	110 ± 4	117 ± 8	146 ± 6^†††^	150 ± 4^†††^	140 ± 4^†††^	147 ± 4^†††^
Total volume excreted (as percent of saline load infused)	91 ± 8	43 ± 7^#^	150 ± 22**	90 ± 7^#^	65 ± 4	60 ± 4	66 ± 9^†††^	59 ± 8
Total sodium excreted (as percent of saline load infused)	92 ± 11	42 ± 6^##^	130 ± 10*	73 ± 7^###^	68 ± 6	81 ± 7^†^	75 ± 15^†††^	42 ± 8#^†^
**%∆PRA**								
180 min (end saline)	− 66 ± 6	− 35 ± 6^#^	− 46 ± 3*	− 35 ± 1^#^	− 57 ± 4	− 64 ± 5*^††^	− 40 ± 3	− 32 ± 3
360 min (end recovery	− 3 ± 8	− 38 ± 5^##^	9 ± 8	− 33 ± 5^##^	− 17 ± 11	− 18 ± 6	− 18 ± 6	− 18 ± 8

%∆PRA; percent change in PRA from baseline; *P < 0.05, **P < 0.01 comparing between intact groups within age, ^#^P < 0.05, ^##^P < 0.01, ^###^P < 0.001 comparing between intact and RDN groups within age, ^†^P < 0.05, ^††^P < 0.01, ^†††^P < 0.001 comparing groups between ages from post-hoc analysis following repeated measures ANOVA. All animals received an isotonic saline vehicle infusion of 12 ml/h containing ^51^Chromium EDTA and para-aminohippuric acid over the day (total volume infused 96 ml) but this has not been included in the total volume/sodium infused and excreted as it does not affect the differences between the treatment groups.

### Saline loading-urine flow and urinary sodium excretion

**Figure 3:**
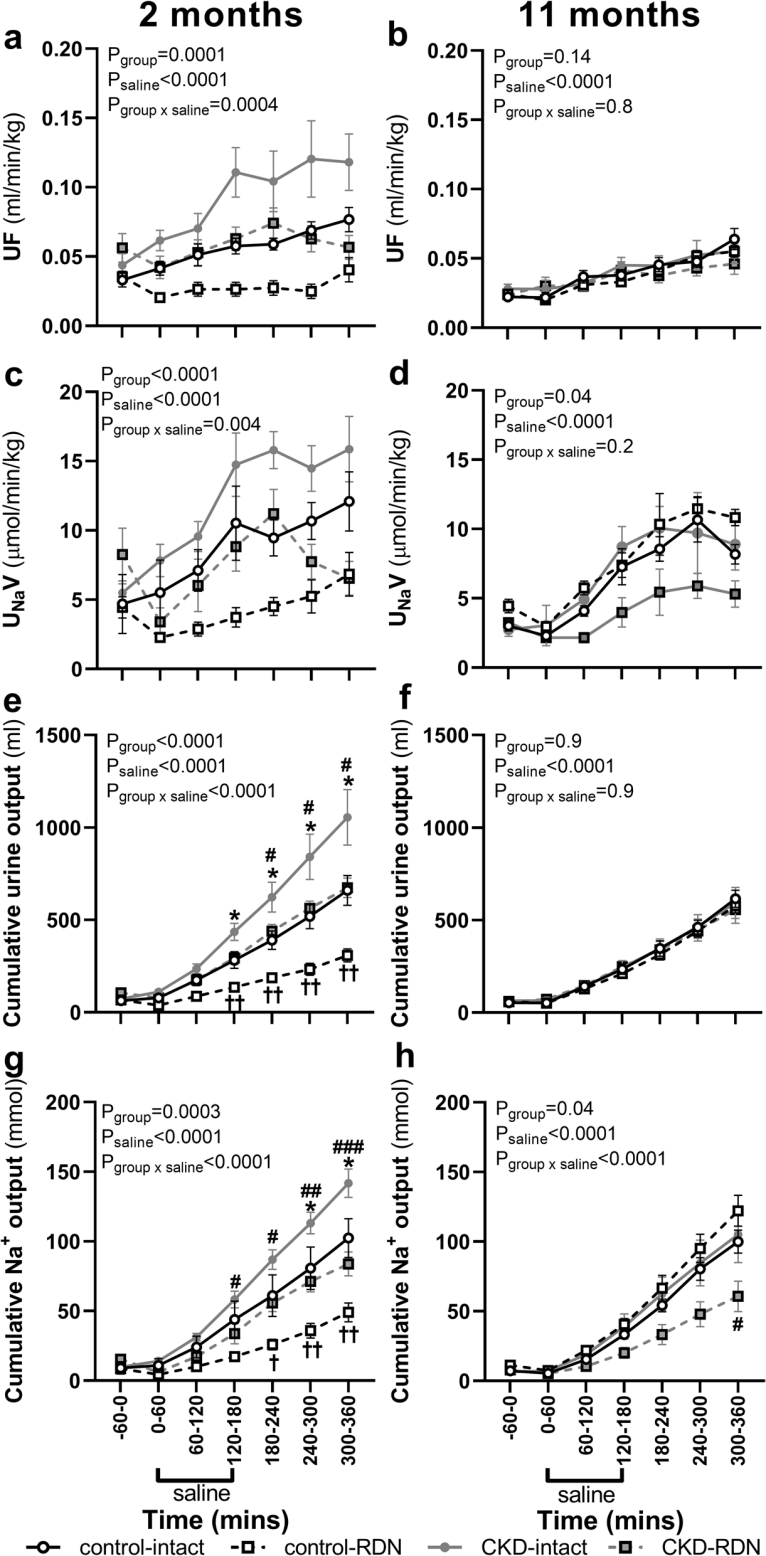
Urine flow, urinary sodium excretion, cumulative urine and sodium output in response to isotonic saline loading. Responses examined in normotensive sheep and sheep with hypertensive chronic kidney disease (CKD) at 2 and 11 months after sham (intact) or renal denervation (RDN) procedure. Data are at baseline; − 60–0 min, during 180 min of saline infusion and during 180 min of recovery post saline infusion. Data are mean ± SE. *P < 0.05 comparing CKD-intact with control-intact, ^#^P < 0.05, ^##^P < 0.01, ^###^P < 0.001 comparing CKD-intact with CKD-RDN, ^†^P < 0.05, ^††^P < 0.01comparing control-intact with control-RDN, from post-hoc analysis following repeated measures ANOVA. *UF* urine flow, *U*_*Na*_*V* urinary sodium excretion.

On the day of saline loading, baseline urine flow and U_Na_V were similar between the groups at both time-points (Fig. 3a–d). There was no difference in body weights of treatment groups (Table 1). All treatment groups received a similar amount of saline load at 2 and 11 months except that owing to being heavier, animals at 11 months received a greater load compared with 2 months (Table 1). An effect of saline loading was observed on urine flow and U_Na_V at both time-points (P_saline_ < 0.0001 for both time-points, Fig. 3a–d). At 2 months after sham procedure, cumulative urine and cumulative sodium output in response to saline loading were greater in CKD-intact group compared with control-intact (urine; ~ 60%, sodium; ~ 40%) but urine and sodium output between CKD and control intact groups were similar at 11 months (Fig. 3a–d). At 2 months after RDN, urine and sodium output in response to saline load were less in both control and CKD RDN groups than their intact counterparts (control; urine: ~ 53%, sodium: ~ 52%, CKD; urine ~ 36%, sodium: ~ 41%, Fig. 3e,g). At 11 months cumulative urine and sodium output were similar between control-intact and control-RDN groups (Fig. 3f,h). Whilst cumulative urine output was similar between CKD-intact and CKD-RDN groups at 11 months after RDN, cumulative sodium output was ~ 42% less in CKD-RDN group compared with CKD-intact at this time-point (Fig. 3h).

The excretion of sodium and volume load differed with time. Expressed as percent of saline load infused, total urine and sodium output at 12 months of age was greater in CKD-intact than control-intact group (urine; ~ 59%, P = 0.002, sodium; ~ 38%, P = 0.04, Table 1) but similar between intact groups at 21 months of age (Table 1). This was because at 21 months compared with 12 months, CKD-intact group excreted less of the infused saline load (urine; ~ 84%, P < 0.0001, sodium; ~ 55%, P = 0.0005, Table 1). At 2 months after RDN, control and CKD groups excreted less of the infused saline load than intact counterparts (control: urine; ~ 48%, P = 0.01, sodium; ~ 50%, P = 0.003, CKD: urine; ~ 60%, P = 0.0009, sodium; ~ 57% P = 0.0003, Table 1). At 11 months after RDN, control-RDN and control-intact groups excreted a similar percent of the infused saline load. In contrast, excretion of the volume component of saline load was similar between CKD-RDN and CKD-intact groups. However, the CKD-RDN group excreted ~ 55% less sodium of the infused saline load than CKD-intact at 11 months after RDN (P = 0.05, Table 1). Compared with 2 months, at 11 months after RDN, total sodium output (percent of saline infused) was ~ 39% more in the control-RDN group and 31% less in CKD-RDN group (control; P = 0.01, CKD; P = 0.05, Table 1).

### Saline loading-renal hemodynamics

On day of saline loading, baseline GFR and renal blood flow were lower in CKD-intact group compared with control-intact at both time-points (GFR; 2 months; ~ 33%, 11 months; ~ 38%, RBF; 2 months; ~ 27%, 11 months; ~ 31%, P < 0.0001 for both and for both ages, Figure S4a–d). RVR was higher in CKD-intact group compared with control-intact at both time-points (2 months; ~ 46%, 11 months; ~ 62%, P < 0.0001 for both ages, Figure S4e–f). Baseline filtration fraction was not different between intact groups at 2 months but was lower in CKD-intact compared with control-intact at 11 months (P = 0.04, Figure S4g,h).

RDN in the control group had no effect on basal renal hemodynamics (Figure S4a–h). In CKD-RDN group, basal GFR and renal blood flow were greater than CKD-intact at 11 months (GFR; ~ 34%, P = 0.02, RBF; ~ 18%, P = 0.004) but not at 2 months after procedure (Figure S4a–d). However, basal RVR was lower in CKD-RDN group compared with CKD-intact at both time-points (2 months; ~ 17%, 11 months; ~ 11%, P < 0.0001 for both ages; Figure S4e,f). Filtration fraction was similar between CKD-intact and CKD-RDN groups at 2 months but was greater in the CKD-RDN group compared with CKD-intact at 11 months after procedure (P < 0.0001, Figure S4g,h). Saline loading had no effect on any of these parameters in any group (Figure S4a–h).

## Discussion

The main findings of this study were that at 2 months after RDN, natriuretic and diuretic responses to isotonic saline loading were blunted in normotensive (control) and in hypertensive CKD sheep compared with intact counterparts. At 11 months after RDN, diuretic and natriuretic responses were restored to intact levels in denervated normotensive sheep whereas in the denervated hypertensive CKD sheep, the natriuretic response to saline loading remained attenuated. This blunted excretion was not associated with changes in filtered load since GFR did not change in response to saline loading. The fall in PRA in response to saline loading was blunted in both RDN groups compared with intact at 2 months after procedure and may have contributed to blunting of sodium and fluid excretion in the RDN groups at this time. Given that limited regrowth of renal nerves occurred in this cohort of hypertensive CKD sheep at 30 months after RDN^28^, it is likely that the reduced ability to excrete a saline load observed in the current study, maybe prolonged in hypertensive CKD-RDN sheep. Overall, these findings demonstrate that renal nerves contribute to renal excretory responses to volume expansion and RDN impairs renal excretory function in response to a volume expansion challenge.

In sheep with hypertensive CKD that had undergone RDN, blood pressure was lower than non-denervated hypertensive CKD sheep, at both 2 and 11 months after RDN. This is consistent with observations in this cohort of sheep at these timepoints (experiments performed on separate days to the current study)^28,29^. The improvements in kidney function with time after RDN are also consistent with previous observations in these animals^28,29^. Additionally, we have demonstrated that the lowering of blood pressure, and improvements in kidney function, are sustained for up to 30 months after RDN in these sheep^28^. Observations of this study are also consistent with evidence from clinical trials that demonstrate that RDN lowers blood pressure and improves kidney function in patients with hypertension and CKD^16,18,19,21–23,30,31^.

Renal sympathetic nerves innervate all segments of the renal tubules and thus influence sodium transport^32^. In this study, in sheep with intact nerves, urine and sodium excretion increased in response to isotonic saline loading at 12 months of age (2 months after sham procedure). Excretion of the fluid/sodium load was 40–60% greater in CKD-intact than control-intact sheep at this age. Natriuresis and diuresis in response to saline loading are also enhanced in spontaneously hypertensive rat (SHR) compared with normotensive Wistar Kyoto counterpart^33,34^, and in patients with essential hypertension compared with normotensive subjects^35,36^. Additionally, in humans and animals with kidney disease associated with reduction in renal mass, the sodium excretory responses to saline loading are also enhanced compared with healthy subjects^37,38^. Compared with normotensive counterparts, elevations in basal RSNA are present in the SHR (demonstrated by direct nerve recording)^39^, and in patients with essential hypertension (demonstrated by increased renal norepinephrine spillover)^40^. There is also evidence for elevated RSNA in chronic kidney disease^41–44^. Although we have not directly measured RSNA in the sheep with hypertensive CKD, we have demonstrated hyperinnervation of renal efferent sympathetic and renal afferent sensory nerves together with enhanced vascular contraction to renal nerve stimulation^28,45^. These provide strong evidence for elevated RSNA in this ovine model of hypertensive CKD. A greater reduction of RSNA during isotonic saline loading associated with higher basal RSNA contributes to enhanced natriuresis and diuresis in SHR^33,34^. Therefore, the enhanced natriuresis and diuresis observed in response to saline loading in 12-month-old hypertensive CKD-intact sheep, maybe associated with greater reduction in RSNA owing to their higher basal RSNA. Renal excretory responses to volume expansion in this study were affected by age and by disease state. In hypertensive CKD-intact sheep at 21 months of age (11 months after sham procedure), sodium and volume excretion in response to saline loading were similar to that of control-intact. This was because, excretion of saline load decreased with age in CKD-intact sheep. In this ovine model, in response to saline loading, in hypertensive CKD sheep, sodium and volume excretion were enhanced at 6 months but attenuated at 5 years of age compared with normotensive sheep^46^. Although those studies were performed in response to a rapid saline load (50 ml/kg over 30 min), and the animals at 5 years were not the same as the ones experimented on at 6 months, the observation indicates a strong effect of advancing age on handling of extracellular fluid volume expansion in hypertensive CKD sheep. Renal sympathetic tone is not affected by ageing in healthy humans^47^, but renal norepinephrine spillover is lower in elderly patients with essential hypertension compared with younger subjects^48^. Additionally, in patients with renal failure, urinary sodium excretion in response to salt loading is attenuated compared with healthy subjects^49^. Therefore, the blunting of renal excretion of saline load in the older hypertensive CKD-intact sheep in this study, maybe associated with a reduction in RSNA with ageing or with loss of kidney function.

Radiofrequency catheter-based RDN blunted natriuresis and diuresis in response to saline loading in normotensive and hypertensive CKD sheep at 2 months after procedure. Acute RDN in SHR also reduced natriuresis and diuresis to volume expansion^33^ and natriuresis and diuresis to saline loading are also blunted in normotensive animals following surgical RDN^50,51^ and following radiofrequency catheter-based RDN^15^. Thus, our observations are consistent with those of others. Although RDN in dogs with obesity induced hypertension lowered blood pressure indicating a role for increased RSNA in obesity induced hypertension^30^, RDN in obese dogs had no effect on sodium excretion in response to an acute saline load^13^ or a sodium rich meal^14^. This suggests that renal nerves may not influence renal sodium excretion in all forms of hypertension. In the present study, at 11 months after RDN, excretion of saline load was similar between control-RDN and control-intact groups. This response is most likely associated with return of renal nerve function in control-RDN sheep, because we have shown that structural and functional regrowth of renal nerves are complete by 11 months after radiofrequency catheter-based RDN in normotensive sheep^25,28^. Return of renal nerve function was further evident in the fact that basal PRA, PRA in response to saline loading, and fluid and electrolyte excretion in response to saline loading, all increased in control-RDN group and were comparable to levels of control-intact, at 11 months after procedure. In CKD-RDN group, the natriuretic response to saline loading remained attenuated and decreased further with time, but the diuretic response was the same as CKD-intact at 11 months. The mechanisms behind the dissociation between natriuretic and diuretic responses in CKD-RDN sheep are unclear. Since radiofrequency catheter-based RDN ablates both the efferent renal sympathetic nerves and the afferent renal sensory nerves, it is likely that removal of renal afferents may have reduced release of arginine vasopressin (AVP) and resulted in greater urine output. We have been unsuccessful in measuring plasma AVP or copeptin levels in response to stimuli that depress these hormonal systems (i.e., saline load) in sheep^15^. Therefore, the role of AVP in saline induced diuresis was not possible to be examined in this study. Renal aquaporin 2 (AQP-2) gene expression is lower in sheep with hypertensive CKD compared with normotensive sheep^52^, and RDN has been shown to reduce AQP-2 protein expression in the rat kidney^53^. Therefore, either reduced AVP or a greater reduction in AQP-2 following RDN (or both) may account for the greater diuresis compared with natriuresis in CKD sheep at 11 months after RDN. In this cohort of hypertensive CKD sheep, at 30 months after RDN, limited regrowth and return of renal nerve function was evident whereas complete restoration was observed in control-RDN sheep^28^. Therefore, limited regrowth and return of nerve function likely underlie the blunted natriuretic response to saline loading in hypertensive CKD sheep at 11 months after RDN. Collectively, these observations indicate that withdrawal of tonic RSNA in response to volume expansion is important in excretion of a sodium and fluid load. Consequently, RDN impairs renal excretory function in response to challenges to extracellular fluid volume homeostasis in both normotensive and hypertensive CKD settings. However, these impairments wane as renal nerve function returns at least in the normotensive animal.

In our previous studies, in response to rapid saline loading, the natriuresis and diuresis in both normotensive and hypertensive sheep, have in part been driven by large increases in GFR^46,54^. In the present study, GFR was unchanged in response to saline loading in all groups at both time-points. In the absence of changes in GFR, increases in natriuresis and diuresis during volume expansion are associated with reductions in fractional reabsorption of water and sodium in the proximal tubules^1^. Tubular sodium reabsorption in the proximal tubules is strongly influenced by regulation of sodium transport by the renin angiotensin system^55^. Suppression of renin release influences the natriuretic response to volume expansion in both normotensive and hypertensive settings^46,56–58^. PRA fell in response to saline loading in all groups in this study. At 2 months after procedure, the fall in PRA was less in CKD-intact compared with control-intact (20%) and was less in RDN groups than intact counterparts (control; 31% and CKD; 11%). Patients with essential hypertension also have a blunted suppression of renin in response to saline loading^56^. In subjects with kidney failure, the fall in PRA in response to high-salt intake was the same as that of healthy subjects^49^. In a study in uninephrectomised rats with hypertension the reduction in PRA in response to high-salt intake was also similar to that of control rats^59^ but in another study in rats with subtotal nephrectomy, the reduction in PRA in response to high-salt intake was greater than control rats^60^. However, in patients with hypertension and CKD, the fall in PRA in response to high salt intake was shown to be blunted compared with healthy controls^61,62^. The mechanisms behind the lesser suppression of renin in the present study are unclear. Increased NaCl delivery to the macula densa activates the tubuloglomerular feedback (TGF) mechanism and simultaneously suppresses renin^63^. We have shown that sensitivity of the TGF mechanism is reduced in hypertensive CKD sheep^64^. A reduction in sensitivity of TGF is usually associated with a decrease in inhibition of renin^65^. Therefore, the blunted fall in PRA, in response to saline loading in the hypertensive CKD sheep, maybe associated with a reduction in sensitivity of the TGF mechanism. RDN also reduces the sensitivity of TGF both acutely and chronically but TGF sensitivity improves overtime following chronic RDN^66,67^. In the denervated sheep, the fall in PRA was blunted in both normotensive and hypertensive groups compared with intact counterparts at 2 months but not at 11 months after RDN. Therefore, a reduction in sensitivity of TGF may account for the blunted fall in PRA in the younger denervated sheep. Conversely, recovery of TGF sensitivity overtime may account for the improvement of the PRA response to saline loading in the older denervated sheep. Logic would suggest that a lesser fall in PRA should be associated with a lesser excretion of sodium and volume load. This was observed in the older hypertensive sheep and the denervated normotensive and hypertensive sheep but not the younger hypertensive sheep. Basal PRA is low in this ovine model of hypertensive CKD. In CKD, PRA has been demonstrated to be low or normal^68^. In the present study, the low PRA may be an appropriate response to the elevated blood pressure in these sheep or as has been shown in other models of reduced renal mass, the reduction in PRA maybe associated with reduced renal renin associated with the reduction in nephron number in this model^60,69,70^. Since basal PRA is low in this ovine model of hypertensive CKD, perhaps it cannot be suppressed too much further with a saline load. It is more likely that a greater withdrawal of RSNA in combination with a fall in PRA (albeit less) contributes to the enhanced natriuresis and diuresis in the younger sheep. As mentioned earlier, renal norepinephrine spillover is lower in older subjects than younger subjects. Therefore, the decrease in sodium/fluid excretion with age in the hypertensive sheep maybe associated with a lesser withdrawal of RSNA combined with a lesser fall in PRA. This same argument may also explain the blunted excretion of the sodium and fluid load in the denervated sheep (i.e., lesser withdrawal of RSNA and lesser fall in PRA). The mechanisms here are unclear but suggest that absence/reduction of renal nerves may reduce modulation of renin inhibition during challenges to extracellular fluid homeostasis, and this may contribute to impairments in renal excretory function.

Limitations of this study are that the animals were undergoing long-term follow-up, thus tissue was not available at the timepoints examined in this study for analysis. Therefore, the mechanisms perturbing responses to isotonic saline loading could not be studied in detail. For the same reason, invasive studies examining tubular sodium reabsorption and TGF could not be performed, therefore the contribution of different segments of renal tubules to sodium excretion are unknown. Another limitation of this study is that studies were only performed in female sheep, therefore the findings may not directly translate to males. Major strengths of this study are that the studies were performed in the same normotensive and hypertensive sheep at both time-points and cardiovascular and renal responses were examined in conscious sheep, so responses are not influenced by anesthesia.

## Conclusion

Catheter-based RDN blunted natriuretic and diuretic response to volume expansion, demonstrating that modulation of tonic RSNA contributes to responses to a physiological challenge to fluid and electrolyte homeostasis. In normotensive sheep, impairments in excretion of saline load waned overtime but the impairment in sodium excretory function persisted in sheep with hypertensive CKD. This indicates that renal excretory function improves with regrowth and return of renal nerve function in normotensive animals. However, limited regrowth and return of renal nerve function may underpin impairments in important regulatory mechanisms during challenges to volume homeostasis in hypertensive CKD sheep. Clinically, a reduced ability to excrete a saline load following RDN may contribute to disturbances in body fluid balance. Therefore, patients with hypertensive CKD may require closer monitoring of body fluid balance over a long time-frame following RDN. Additionally, denervated patients may also need closer monitoring when undergoing any surgical procedures that require fluid resuscitation therapy.

## Methods

The datasets generated during and/or analyzed during the current study are available from the corresponding author on reasonable request.

All experiments were approved by an Animal Ethics Committee of Monash University and performed in accordance with the guidelines of the National Health and Medical Research Council of Australia (MARP#2016/182) and all methods are reported in accordance with ARRIVE guidelines. All procedures these animals underwent have been described in detail elsewhere^28,29^. Following an acclimatization period of 7 days, in pure-bred merino pregnant ewes carrying female fetuses, anesthesia was induced (20 mg/kg thiopentone sodium into ewe's jugular vein) on gestation day 100 ± 1 and following endotracheal intubation, anesthesia was maintained during surgery with isoflurane (1.5–2% in oxygen). Prophylactic antibiotic was administered to the ewe at surgery (Austrapen (ampicillin), 20 mg/kg, i.v. (jugular vein)) and for 3 days after surgery. Analgesia was administered to both ewe and fetus at sites of incisions (Marcaine 0.5% with Adrenalin; 20 mg/kg to ewe and 2 mg/kg to fetus) and further provided to the ewe via a transdermal fentanyl patch (Durogesic, 75 µg/h). Following abdominal incision, the uterus was manipulated, and lamb gently manipulated out of the uterus. An incision was made over the left flank of the fetus and the left kidney was exposed and cleared of surrounding fat. Then, unilateral nephrectomy was performed by ligating the left renal artery, vein and ureter and excising the left kidney (CKD; N = 14). In some fetuses a sham procedure was performed (control; N = 13). where the left kidney was exposed and gently manipulated, but not excised. Only female lambs were used in this study as insertion of a bladder catheter on multiple occasions is easiest performed in female sheep. At 5 months of age, surgery was performed to exteriorize carotid arteries into skin loops to enable easy access for insertion of catheters as previously described^28,29^.

### Radiofrequency catheter based renal denervation

In these sheep, arterial pressure and kidney function were assessed at 6 months of age to establish baseline and these data have been previously reported^71^. At 10 months of age, animals were randomly assigned to receive either RDN procedure (six, two-minute radiofrequency ablations per artery) or sham-procedure using the *Symplicity Flex* catheter (*Symplicity*, Medtronic Ardian Inc, CA) as previously described^29^. This resulted in four groups; control-RDN; N = 7, CKD-RDN; N = 7, control-intact; N = 6 and CKD-intact; N = 7.

### Experimental protocol

Each sheep underwent multiple studies at 2, 5, 11 and 30 months after RDN addressing various research questions for which some data have been reported^28,29^. Data reported in this study include the following two protocols performed at 2 (12 months of age), and repeated at 11 (21 months of age) months after RDN or sham procedure in the same sheep: (1) time control study and (2) a study in which the response to isotonic saline loading was examined. All experiments were conducted in unanesthetized sheep and 2–3 days separated each study. In between study timepoints, animals were housed on pasture. Prior to a study period, animals were housed in an indoor barn where they had free access to hay and chaff (cut-grass) and water. After acclimatization to indoor conditions for 7 days, animals were housed individually in cages and maintained on the diet of hay and chaff (cut-grass) and had access to water ad-libitum.

### Arterial and venous cannulations

After 5 days of housing in cages, under local anesthesia (subcutaneous, 20 mg/kg, Lignocaine 20), cannulas were inserted into each carotid artery to measure mean arterial pressure (MAP) and heart rate, and for blood collection and into each jugular vein for infusion purposes. For renal function studies, a Foley catheter (Size 10 or 12) was inserted into the bladder under local anesthesia (Xylocaine Jelly, 2%) on the morning of the experiment.

### Time control-series (vehicle infusion)

Glomerular filtration rate (GFR) was assessed by clearance of ^51^Chromium ethylenediaminetetraacetic acid (^51^Cr EDTA) and renal blood flow (RBF) by the clearance of para-aminohippuric acid (PAH), as previously described^28,29,71^. On the day of experiment, after a 1 h equilibration period, urine samples were collected every 30 min, with an arterial blood sample (5 ml) collected mid-point for the duration of 7 h. BP and heart rate were acquired every 10 s and averaged over 10 min. The 10 min averages were averaged over 60 min and that hourly average is presented in the figures. Renal vascular resistance (RVR) and filtration fraction were calculated as previously described^71^.

### Isotonic saline loading series

Renal hemodynamic, urinary excretion and cardiovascular (MAP and heart rate) responses to 180 min of isotonic saline (0.9% sodium chloride, 154 mmol/l, Baxter Healthcare, USA) were examined on a separate day. Similar to the time-control series, following a 1-h equilibration period, cardiovascular and renal function were measured over 60 min to establish baseline before commencement of isotonic saline infusion at 0.13 ml/kg/min equating to 20 µmol Na^+^/kg/min for 180 min (i.v) followed by a 180 min period of recovery, with blood and urine samples collected as described above. A 5 ml blood sample was also collected at end of baseline, saline infusion and recovery period for analysis of plasma renin activity (PRA) via radio-immunoassay (ProSearch International). Urine samples were analyzed for sodium concentration (Beckman Coulter, Monash Medical Centre) and sodium excretion calculated.

### Statistical analysis

All values are presented as mean ± SEM. Statistical analysis was performed using GraphPad software (GraphPad Prism 9.0.0, version 1 for Windows, GraphPad Software Inc, USA, Prism-GraphPad), with the level of significance set at P ≤ 0.05. Data were tested for normality using a Shapiro–Wilk test and were determined to fit Gaussian distribution. Raw data for cardiovascular and kidney function variables were analyzed separately for the animals at 2- and 11-months post RDN/sham procedure. A repeated measures analysis of variance (ANOVA) was performed within ages examining the effects of two factors; group (P_group_; intact or RDN) and time (P_time_) and their interactions for time-control series data. Similarly, a repeated measures ANOVA was performed to examine effect of saline loading within ages examining the effects of two factors; group (P_group_; intact or RDN) and saline (P_saline,_ before and after saline loading) and their interactions. Data for the load of saline infused and excreted were analyzed using a repeated measures ANOVA with factors group (P_group_; intact or RDN) and age (P_age_; 2 and 11 months) and their interactions. Multiple comparisons test was performed to compare the differences in responses between groups and ages as appropriate.

## Supplementary Information

Supplementary Information 1.
