# Supplementary material for: Blunted natriuretic response to saline loading in sheep with hypertensive kidney disease following radiofrequency catheter-based renal denervation
Source: Sci Rep. 2021 Jul 20;11:14795. doi: 10.1038/s41598-021-94221-5 (PMC8292431; doi:10.1038/s41598-021-94221-5)
Supplement: Supplementary file 1 — Supplementary Information 1. [file 41598_2021_94221_MOESM1_ESM.docx]

**Blunted natriuretic response to saline loading in sheep with hypertensive kidney disease following radiofrequency catheter-based renal denervation**

^1^Reetu R Singh*, ^1^Zoe McArdle, ^1^Harshil Singh, ^2^Lindsea C Booth, ^2^Clive N May, ^3^Geoffrey A Head, ^4^Karen M Moritz, ^5^Markus P Schlaich, ^6^Kate M Denton

^1^Cardiovascular Program, Monash Biomedicine Discovery Institute and Department of Physiology, Monash University, Melbourne, Australia, ^2^The Florey Institute of Neuroscience and Mental Health, The University of Melbourne, Parkville, Australia, ^3^Baker IDI Heart and Diabetes Institute, Melbourne, Australia, ^4^School of Biomedical Sciences, The University of Queensland, Brisbane, Australia, ^5^School of Medicine and Pharmacology-Royal Perth Hospital Unit, University of Western Australia, Australia

**Running title: Renal denervation and volume expansion**

**Address for correspondence:**

Reetu R Singh (PhD)

Cardiovascular Program, Monash Biomedicine Discovery Institute and Department of Physiology

Monash University

Clayton

VIC, 3800

Australia

Ph: 613-99052285

Fax: 613-99052547

Email: Reetu.Singh@monash.edu

**Data Supplement Figures**

**Figure S1**


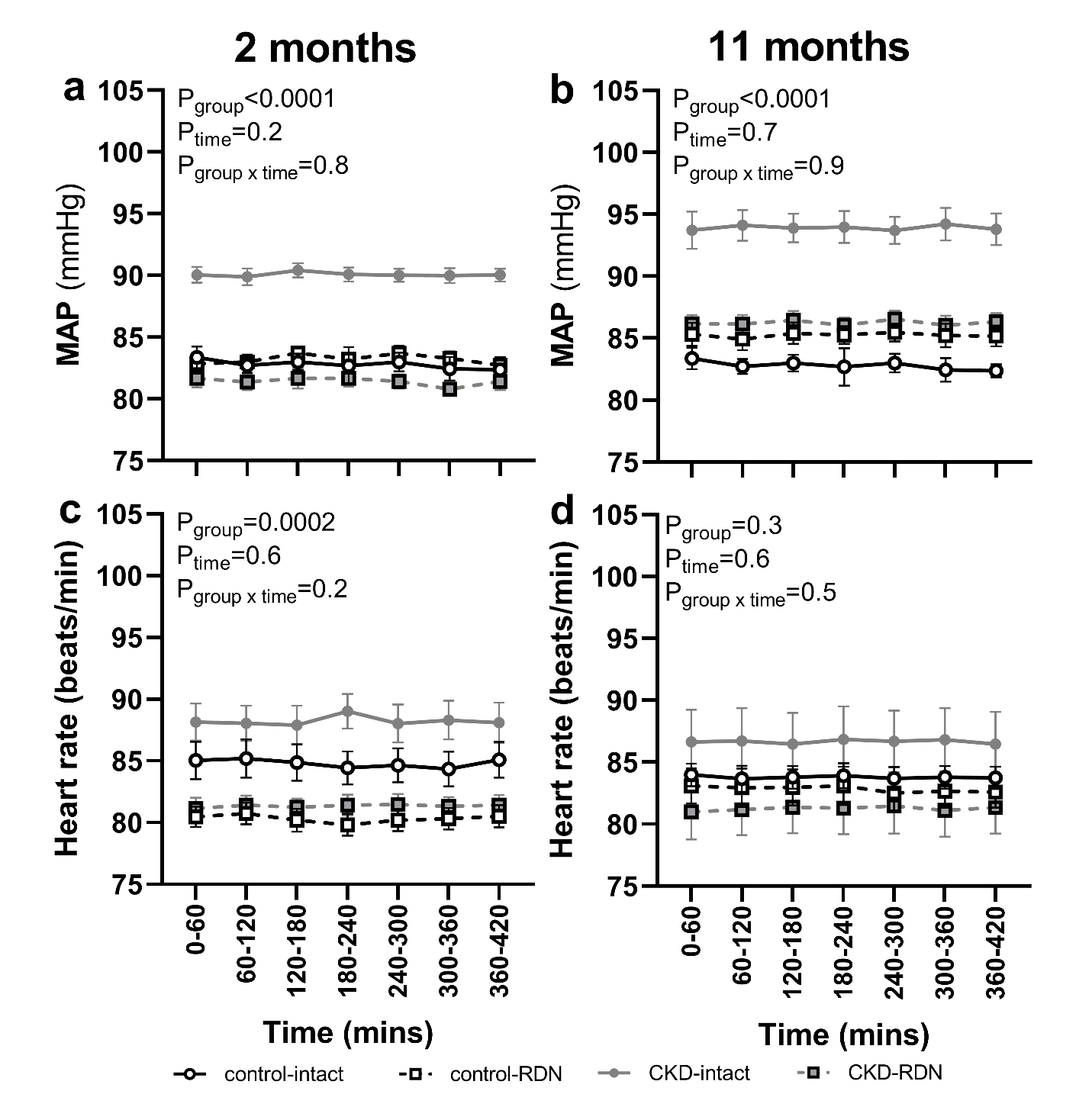


**Figure S1**: Mean arterial pressure (MAP) and heart rate during 7 hours of vehicle infusion (time-control) at 2 and 11 months after sham (intact) or renal denervation (RDN) procedure in normotensive sheep and in sheep with hypertensive chronic kidney disease (CKD). Data are mean± S.E.

**Figure S2**


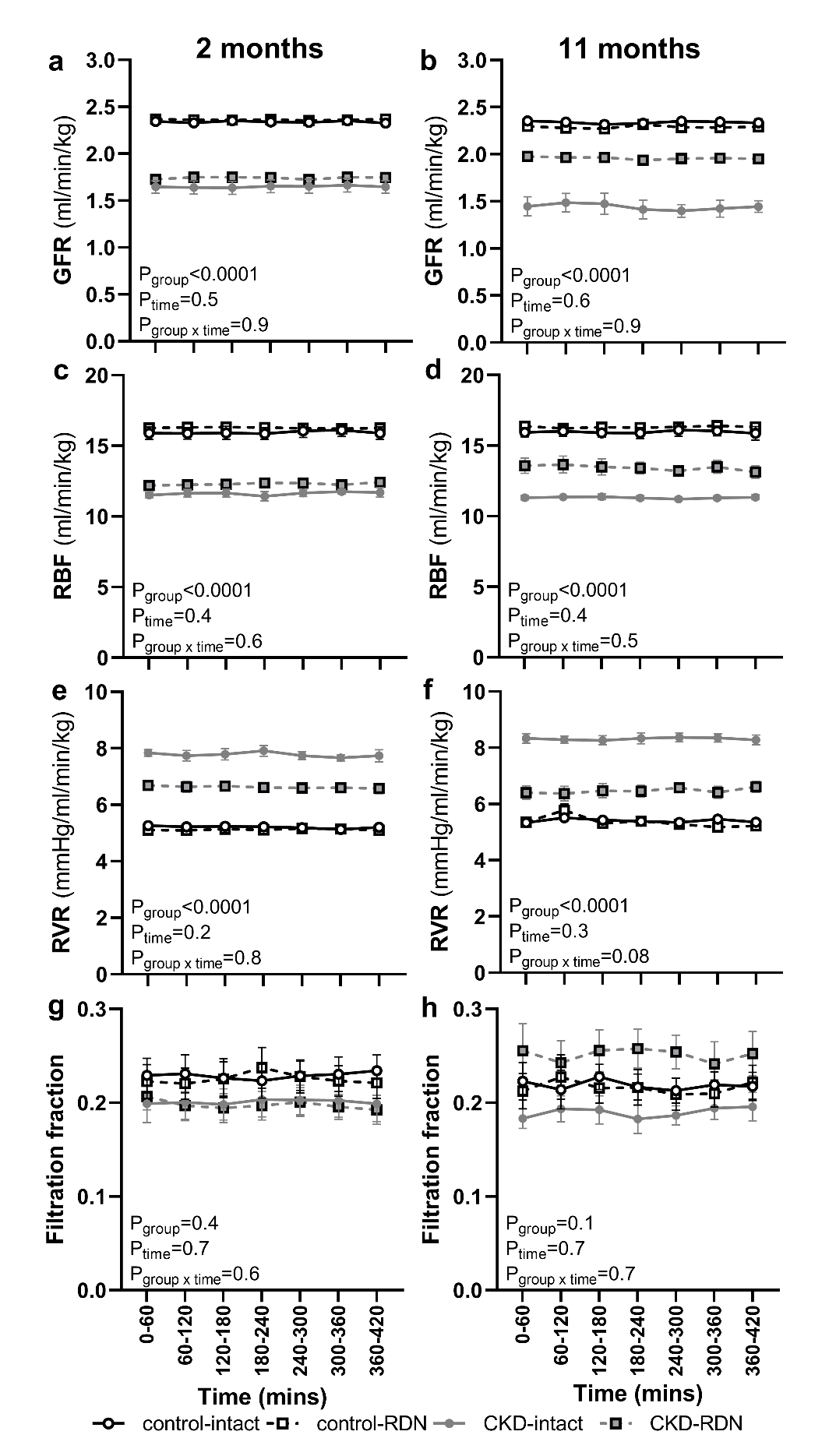


**Figure S2**: Kidney hemodynamics during 7 hours of vehicle infusion (time-control) at 2 and 11 months after sham (intact) or renal denervation (RDN) procedure in normotensive sheep and in sheep with hypertensive chronic kidney disease (CKD). Data are mean± S.E. GFR; glomerular filtration rate, RBF; renal blood flow, RVR; renal vascular resistance.

**Figure S3**


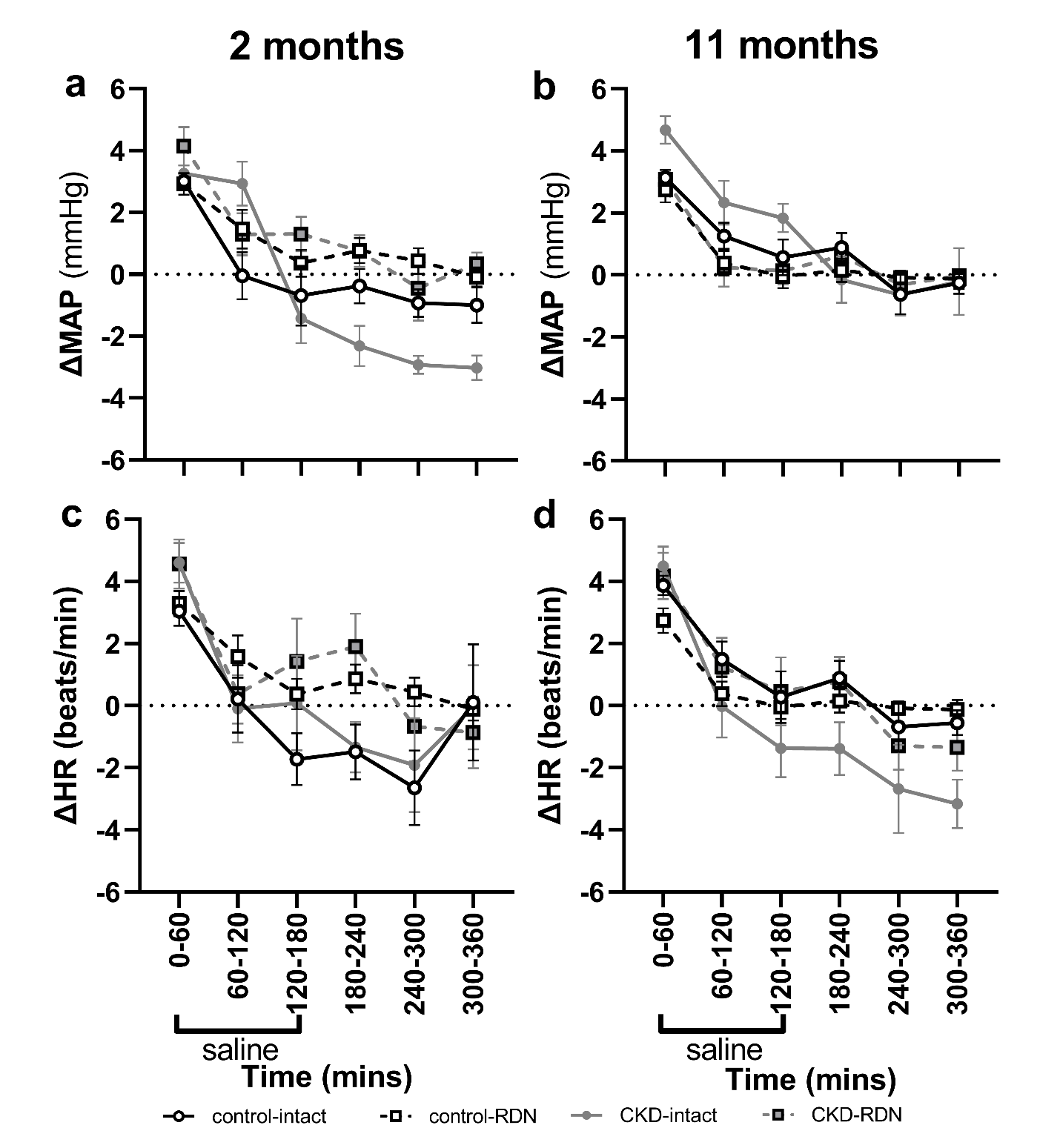


**Figure S3:** **Change from baseline in mean arterial pressure (MAP) and heart rate during saline loading and recovery period**. Data are mean± S.E.

**Figure S4**


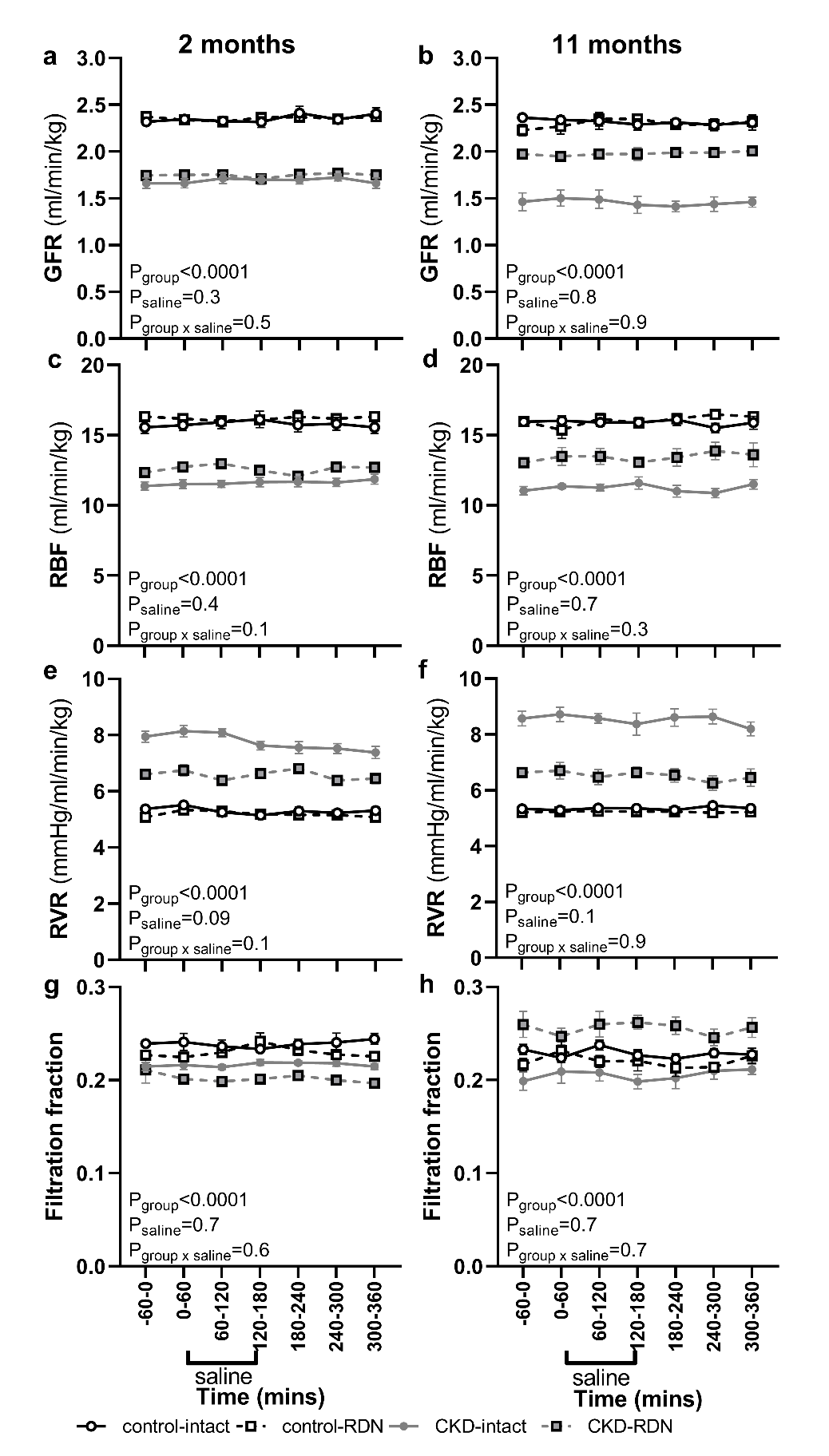


**Figure S4: Kidney hemodynamics in response to isotonic saline loading at 2 and 11 months after sham (intact) or renal denervation (RDN) procedure in normotensive sheep and in sheep with hypertensive chronic kidney disease (CKD).** Data are mean ± S.E. Baseline; -60 mins. GFR; glomerular filtration rate, RBF; renal blood flow, RVR; renal vascular resistance.
